# Supplementary material for: D-amino acid substitution and cyclization enhance the stability and antimicrobial activity of arginine-rich peptides
Source: Microbiology (Reading). 2026 Feb 4;172(2):001657. doi: 10.1099/mic.0.001657 (PMC12873548; doi:10.1099/mic.0.001657)
Supplement: Uncited Supplementary Material 1. [file mic-172-01657-s001.pdf]

Supplementary Material

D-amino acid substitution and cyclisation enhance the stability and antimicrobial activity of arginine-rich peptides

Bruno Mendes[1], Valeria Castelletto[2], Ian W. Hamley[2] and Glyn Barrett[1*]

[1]*School of Biological Sciences, University of Reading, Whiteknights, Reading, RG6 6AH, U.K.*

[2]*School of Chemistry, Pharmacy and Food Biosciences, University of Reading, Whiteknights, Reading RG6 6AD, U.K.*

[3]*Quantum Detectors, Oxford, OX11 0QX, U.K.*

Supplementary table 1. Sequence and molecular mass of potential antimicrobial peptide candidates.

Peptide	Sequence	Molecular mass (Da)
**R4A4**	RRRRAAAA	927.063
**R4V4**	RRRRVVVV	1039.278
**R4F4**	RRRRFFFF	1231.452
**R5F5**	RRRRRFFFFF	1534.815
**R6F6**	RRRRRRFFFFFF	1838.178
**R2F4R2**	RRFFFFRR	1231.452
**PR4F4**	PRRRRFFFF	1328.568
**RRARSAVAS**	RRARSAVAS	973.086

Supplementary table 2. Sequence and molecular mass of chemically modified peptides.

Peptide	Sequence	Molecular mass (Da)
**R4-C16**	RRRR-NH-palmitoyl	866.193
**R4F4-C16**	RRRRFFFF-NH-Palmitoyl	1454.897
**D-R4F4**	rRrRfFfF	1231.452
**CP-R4F4**	(RRRRFFFF)	1213.437

A

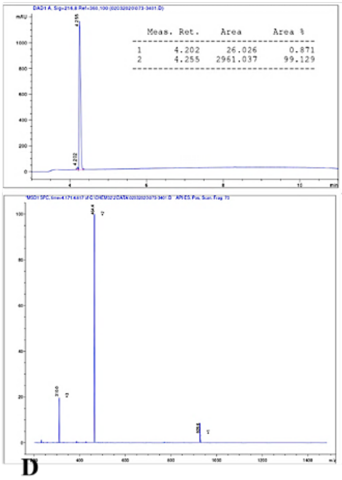

B

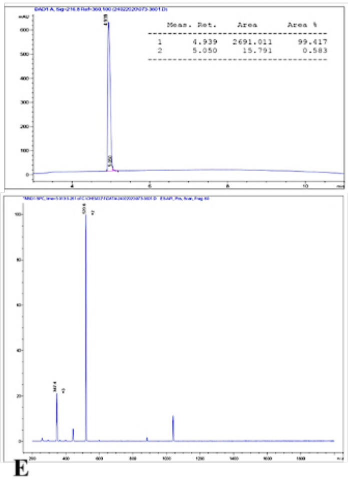

C

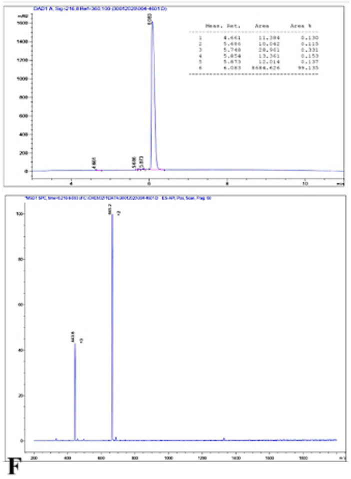

D

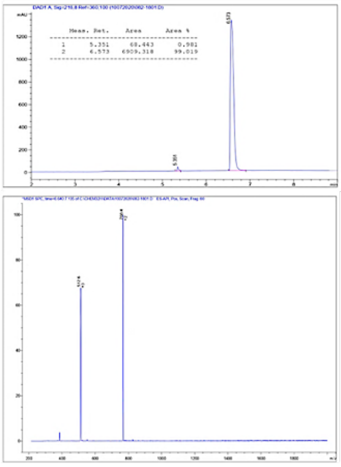

E

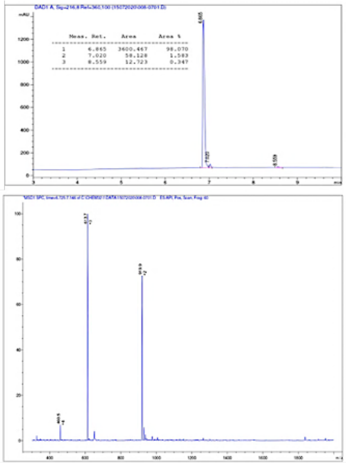

F

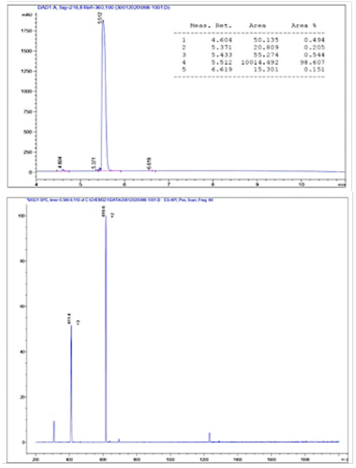

G

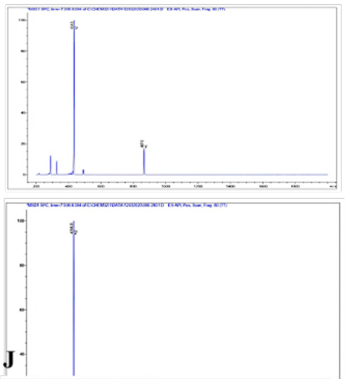

H

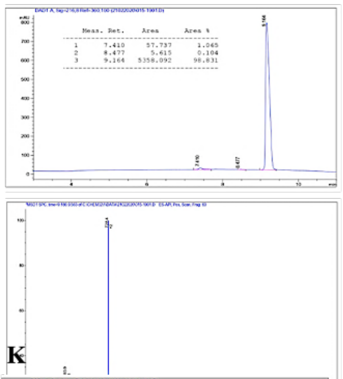

I

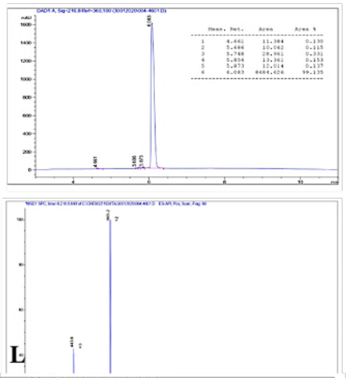

J

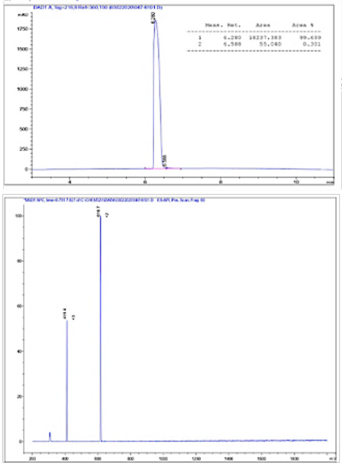

K

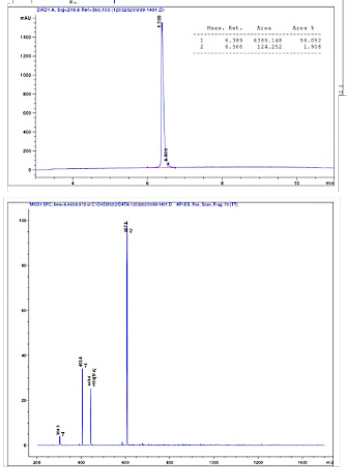

L

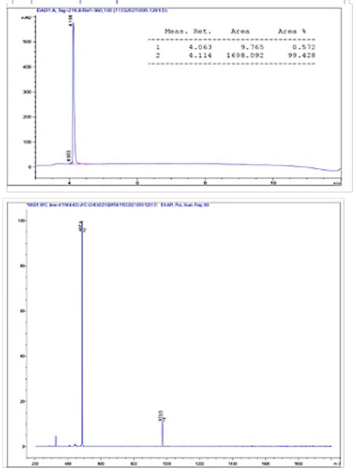

Figure S1. Purity determination and characterization of synthetic peptide by HPLC and mass spectrometry, respectively. Peptide profiles of (A) R4A4, (B) R4V4, (C) R4F4, (D) R5F5, (E) R6F6, (F) R2F4R2, (G) R4-C16, (H) R4F4-C16, (I) PR4F4, (J) D-R4F4, (K) CP-R4F4 and (L) RRARSAVAS showing >95% purity levels. The peaks of retention times and the areas representing purity levels are highlighted in the upper graphs. The elution of synthetic peptides was evaluated using a Kinetex XB-C18 column (100 Å, 4.6 x 50 mm) with a gradient ranging from 10% to 90% acetonitrile over 10 min. Bottom graphs show mass-to-charge ratio ions of products confirmed by mass spectrometry.

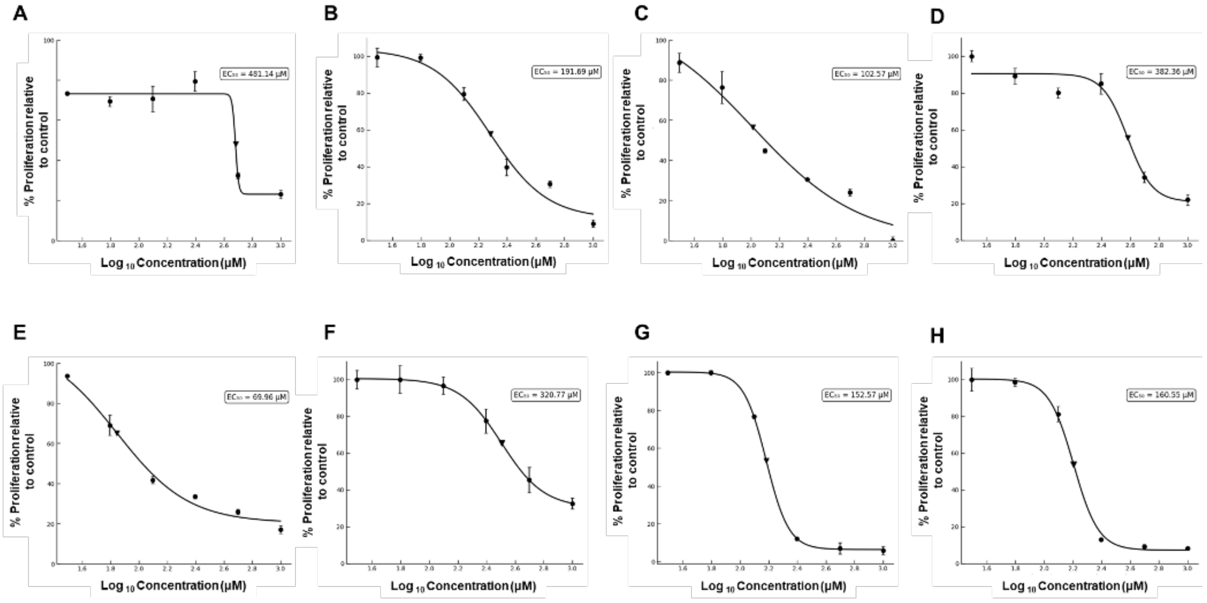

Figure S2. Sigmoidal dose–response curves from the MTS assay showing the EC_{50} values of L929 cells exposed to increasing concentrations of peptides. Approximately 10^5 fibroblast cells were seeded into 96-well plates and incubated for 24 h at 37 °C and 5% CO_2 with peptides: (A) R4F4, (B) R5F5, (C) R6F6, (D) R2F4R2, (E) R4F4-C16, (F) PR4F4, (G) D-R4F4, and (H) CP-R4F4. % proliferation was calculated relative to untreated controls using the MTS assay. The lipidated peptide (E) exhibited a lower EC_{50} (69.96 μM) compared to its non-lipidated analogue (A) EC_{50} (481.14 μM).
